# A Computational Pitting Corrosion Model of Magnesium Alloys

**DOI:** 10.3389/fbioe.2022.887444

**Published:** 2022-05-13

**Authors:** Chia-Jung Chang, Chih-Han Chang, Tin-Kan Hung

**Affiliations:** ^1^ Department of Biomedical Engineering, National Cheng Kung University, Tainan, Taiwan; ^2^ Department of Bioengineering, University of Pittsburgh, Pittsburgh, PA, United States

**Keywords:** computational simulation, magnesium alloy, micro-CT, Monte Carlo method, pitting corrosion

## Abstract

Controlling the corrosion rate of implants to maintain mechanical properties during tissue healing is significant in developing magnesium alloy implants. In addition to surface treatment and material properties, the study of geometric alteration and mechanical strength are also vital for implant development. In this study, we developed a three-dimensional model for semi-autonomous computational pitting corrosion. It is based on the Monte Carlo method, modeling magnesium alloy implants toward clinical application. The corrosion probability is based on the number of exposed surfaces to saline and the oxidation characteristics of the elements. The computational results are well compared with the experimental measurement using micro-computed tomography (micro-CT) in 500 h. Subsequently, the computational analysis is extended to 3,000 h of corrosion analysis. The 3D model appears promising to assist the development of biodegradable implants.

## Introduction

Magnesium (Mg) alloys are prospective biodegradable materials for medical applications in recent years. The alloys have low density, biocompatibility, and mechanical and biodegradable properties comparable to human bones (Soya et al., 2014; Živić et al., 2014; Bowen et al., 2018). Magnesium bone implants can minimize the effects of stress shielding in traditional metallic implants (Xu et al., 2007). Biodegradable magnesium alloy stents have a better bracing force and tissue compatibility than absorbable polymer stents (Li et al., 2019). However, there remains difficulty to control the corrosion rate of implants. Magnesium alloys corrode too fast for maintaining mechanical properties, and the implant function before tissue regeneration and healing are well developed (Song and Song, 2007; Kirkland et al., 2012; Kirkland and Birbilis, 2014).

Bioengineering strategies for controlling the corrosion rate of Mg alloys were discussed in many studies on surface modification (Li et al., 2013; Córdoba et al., 2016), element alloying (Hort et al., 2010; Jin et al., 2015), and microstructure modification (Wu et al., 2017). Improvements in corrosion resistance and implant surfaces remain the main tasks in research and development. The spatial characteristics are also important factors affecting the mechanical behaviors of the implant. Pitting corrosion could cause stress concentration and crack formation (Suresh, 1998; Gu et al., 2010), leading toward peeling off, fragmented fracture (Li et al., 2016), and the collapse of implants.

With advances in technology and computing power, computational models are becoming practical for analyzing complex corrosion phenomena. Quite a number of studies were focused on modeling pitting corrosion and were well compared in review (Jafarzadeh et al., 2019). Non-autonomous models employed the classical transport equation for numerical solutions to transport kinetics in the electrolyte (Sharland et al., 1989; Krouse et al., 2014). They provided a good framework, but tracking pit growth is addressed as a separate part that increases the modeling complexity (Jafarzadeh et al., 2019). Autonomous models such as cellular automata (CA) techniques (Cui et al., 2019), the finite volume method (FVM) (Scheiner and Hellmich, 2009), peridynamic (PD) formulations (Jafarzadeh et al., 2018), and phase-field (PF) models (Tsuyuki et al., 2018) were focused on calculating the motion of corrosion front directly. Nonetheless, there remained many limitations with various autonomous models (Jafarzadeh et al., 2019). The CA model had difficulty to calibrate, and its grid size and time are not physical parameters (Stafiej et al., 2013; Pérez-Brokate et al., 2016), while the FVM did not capture mechanical damage and diffuse the corrosion layer (Jafarzadeh et al., 2019); the PD model had nonlocal effects at boundaries (Le and Bobaru, 2018), and the damage evolution in PF models appeared limited because of its dependency in selecting the energy functional (Geelen et al., 2019) with the dense mathematical framework.

In this study, a semi-autonomous computational model based on the Monte Carlo process is developed for pitting corrosion, with elements having different material characteristics and the surrounding condition. Effort is also made on the oxidized surface characteristics for the specimen with pitting corrosion. The computational results are compared with the experimental 3D images reconstructed by micro-computed tomography (micro-CT, μCT) for 500 h experiments, making the combined use of computation and experiment feasible to project long-term corrosion development.

## Materials and Methods

### Corrosion Experiment

Corrosion of Mg alloy, AZ61, in 0.90% saline was studied individually using 11 specimens of size 15 mm × 15 mm × 2 mm. Weight compositions (wt%) of the AZ61 alloy are listed in Table 1. With initial 570 mm^2^ surface area (*S*_*O*_), each specimen was immersed and suspended in 114 ml of solution at room temperature (25°C ± 2°C). Based on the ASTM31-12a protocol (NACE, 2012), it was dried every 25 h for micro-CT scanning and mass measurement. The saline was then refreshed for the next 25 h of corrosion measurement. Including the initial scan, 21 scans were obtained for each experiment over a period of 500 h. They were obtained using Bruker Micro-CT Sky Scan 1,076 with the following parameters: source voltage = 100 kV, source current = 100 μA, filter = Al 1.0 mm, exposure time = 316 ms, image pixel size = 34.44 μm, and rotation step = 1.000°. Sky Scan software and NRecon software were employed for volumetric reconstruction of each scan measurement, resulting in a total of 22 × 21 three-dimensional (3D) imaging.

**TABLE 1:** Compositions (wt%) of AZ61 in this study.

	Al	Zn	Mn	Cu	Fe	Si	Ni
AZ61	6.51	1	0.24	0.0013	0.0032	0.014	0.00061

The micro-CT scans the specimen mass at each scanning stage, which was calculated from the volume multiplied by its initial density of 1.7 g/cm^3^. The mass loss rate $\begin{array}{c} .\\ \mathrm{ML} \end{array}$ per total surface (*S*_*O*_) per hour (mg/mm^2^ h.) is calculated by the following equation:

$$\begin{array}{c} .\\ \mathrm{ML} \end{array}=\frac{M_{o}–M_{N}}{S_{o}\cdot T}\;$$ (1)

where M_O_ (about 760 mg) is the initial mass measured by a microbalance, *M*_*N*_ (mg) is the calculated mass of the specimen at the *N*th scan *via* the measured volume from micro-CT, and *T* is the submerged time in saline.

### Corrosion Simulation

Modeling of the Mg alloy specimen begins with setting uniform hexahedral elements. The corrosion probability (*CP*_*i*_) for each micro element is assessed at each time step (Δt). Its value for the *i*th element is based on two factors: the expose attribute (*EA*_*i*_) and the oxide attribute (*OA*_*i*_). The *EA*_*i*_ value indicates how many surfaces of an element are exposed to saline (Kelly et al., 2002). A hexahedral element has six surfaces; thus, six chances in contact with saline solution are possible. Initially, all the inner elements are not in contact with saline; *EA*_*i*_ is zero in the computational algorithm. For elements on the top and bottom surfaces and four sides, *EA*_*i*_ is set to one; on the edge, *EA*_*i*_ = 2 and *EA*_*i*_ = 3 for the corner element. Song and Atrens (1999) reported that the inhomogeneous property on the oxide layer and different corrosion potential between the oxide layer and the Mg alloy matrix inside can cause pitting corrosion. To simulate the phenomena, the corrosion speed of an element is indicated by its *OA*_*i*_ value. The code of simulation model is written in Matlab, and the output data are stored as a txt file or an .inp file, which is suitable for several numerical analysis software.

Corrosion rates of the Mg oxide surface are much lower than those of the inner alloy; they are needed for assessing *OA*_*i*_ values. Because the corrosion rate of the interior AZ61 Mg alloy element in saline is 13.7 times faster than that of the external oxide layer elements estimated from the experimental data of Wu et al. (2015) and Tkacz et al. (2016), the *OA*_*i*_ value of the oxide layer is assigned to 0.07 when *OA*_*i*_ = 1 is set for micro elements of intact magnesium. However, *OA*_*i*_ values for the spotted microporous surface are not known *a priori* for a specimen. To identify the locations of initial porous elements on the oxide surface, the pit distribution of a specimen measured at the first 200 h is used for specifying *OA*_*i*_ values on the surface. The final selection of *OA*_*i*_ values between 0.4 and 0.6 was further assessed by trial and error *via* the first 200 h of simulation. This step leads to a proper comparison between simulation and experiment. The combined effect of *OA*_*i*_ and *EA*_*i*_ is expressed as the corrosion attribute (*CA*_*i*_):

$$CA_{i}\mathrm{=E}A_{i}\cdot OA_{i}$$ (2)

The corrosion probability (*CP*_*i*_) of the *i*th element is determined as follows:

$$CP_{i}=\frac{\mathrm{ML}}{M_{e}}\cdot \frac{CA_{i}}{\sum_{\mathrm{i=1}}^{n}CA_{i}}$$ (3)

where the ratio of mass loss ( $\mathrm{ML=}\overset{˙}{M_{e}}\cdot S\cdot \mathrm{\Delta t}$ ) to element mass $\left(M_{e}\right)$ indicates the number of elements being corroded at the time step. The ratio of *CA*_*i*_ to the summation of *CA*_*i*_ values of all the n-elements stands for the affecting corrosion rate of each element. Notice that for intact magnesium micro elements, *CP*_***i***_ remains nil as *OAi* = 0. They will not be corroded until they are in contact with saline through pitting surfaces. The increase in the pitting surface with saline could increase the corrosion speed with time.

A Monte Carlo (Sharland et al., 1989) algorithm is employed to randomly select *ML/M*_*e*_ (number of elements) elements to be corroded and those elements would become void in the computational model at the next time step. The algorithm involves generating a random number for each element to compare *CP*_***i***_ values to determine if this element is corroded and removed. The procedure can be repeated N time steps to relieve elements (N is the number of cycles of corrosion, which can be defined according to user needs, each cycle is default to 25 h). The results of the first 500 h are obtained and compared with the experimental results, and the total simulation time was extended to 3,000 h in this study.

### Image Processing and Quantization

To identify the corrosion characteristics, image processing procedures are employed using Matlab to assess the pitting characteristics. Both the micro-CT images and simulation results are converted to 3D models in STL files. To obtain the pitting maximum depth, the maximum depth of each corrosion pit is calculated by the difference between the maximum and minimum of *z* as shown in Supplementary Figure S1 in the Appendix. To obtain the number of corrosion pits and the projected corrosion area on the surface, the cross sections from the 3D model are exported for morphological feature recognition. After using the Canny method (Canny, 1986) to detect the edge of the pit pattern, the enclosed region from the pits is filled for distinguishing the corroded and non-corroded elements as shown in Supplementary Figure S2.

## Results

**FIGURE 1:**
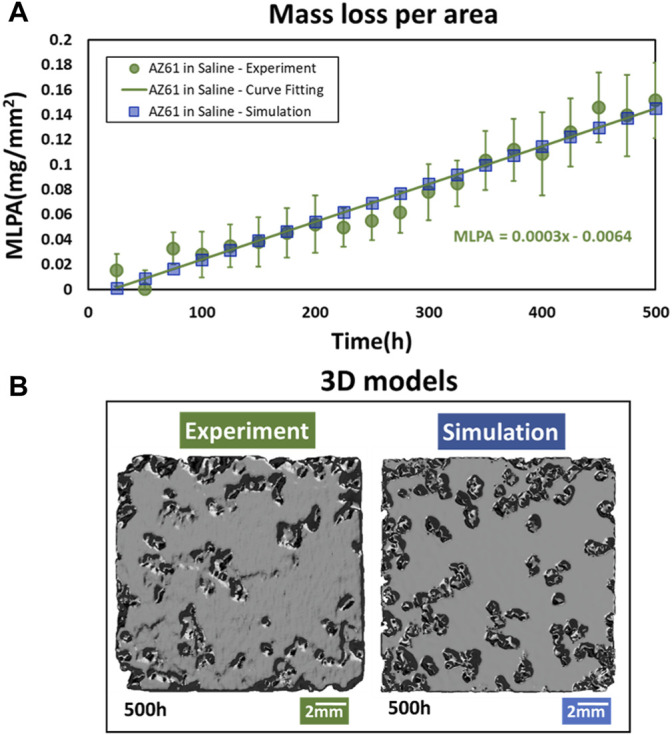
Comparison of overall AZ61 corrosion in simulation and experiment. **(A)** Mass loss per area of experimental specimens and simulation model at different corrosion times. **(B)** 3D graphics.

The experimental results of mass loss with the corrosion time for AZ61 specimen in saline are presented in Figure 1A. Each I-bar represents micro-CT measurements of 11 specimens with a mean value indicated by a circular point. The mass loss per surface area (2 mm × 15 mm × 15 mm) can be fitted by Mass Loss Per Area(MLPA)=0.003T in 500 h. The squares in the figure are the results of simulation; they are in good agreement with the mean values of experimental measurements. 3D graphics in Figure 1B are *via* Autodesk Fusion 360 for making magnesium alloy rendering effects.

Computational results are obtained after identifying the effects of mesh sizes and time steps. The MLPA calculated from simulation is based on four sizes (0.250, 0.175, 0.135, and 0.125 mm) of hexahedral elements. As shown in Supplementary Figure S5, the difference between element sizes of 0.135 and 0.125 mm is under 1.5%, and the micro element of 0.125 mm is used for this study.

**FIGURE 2:**
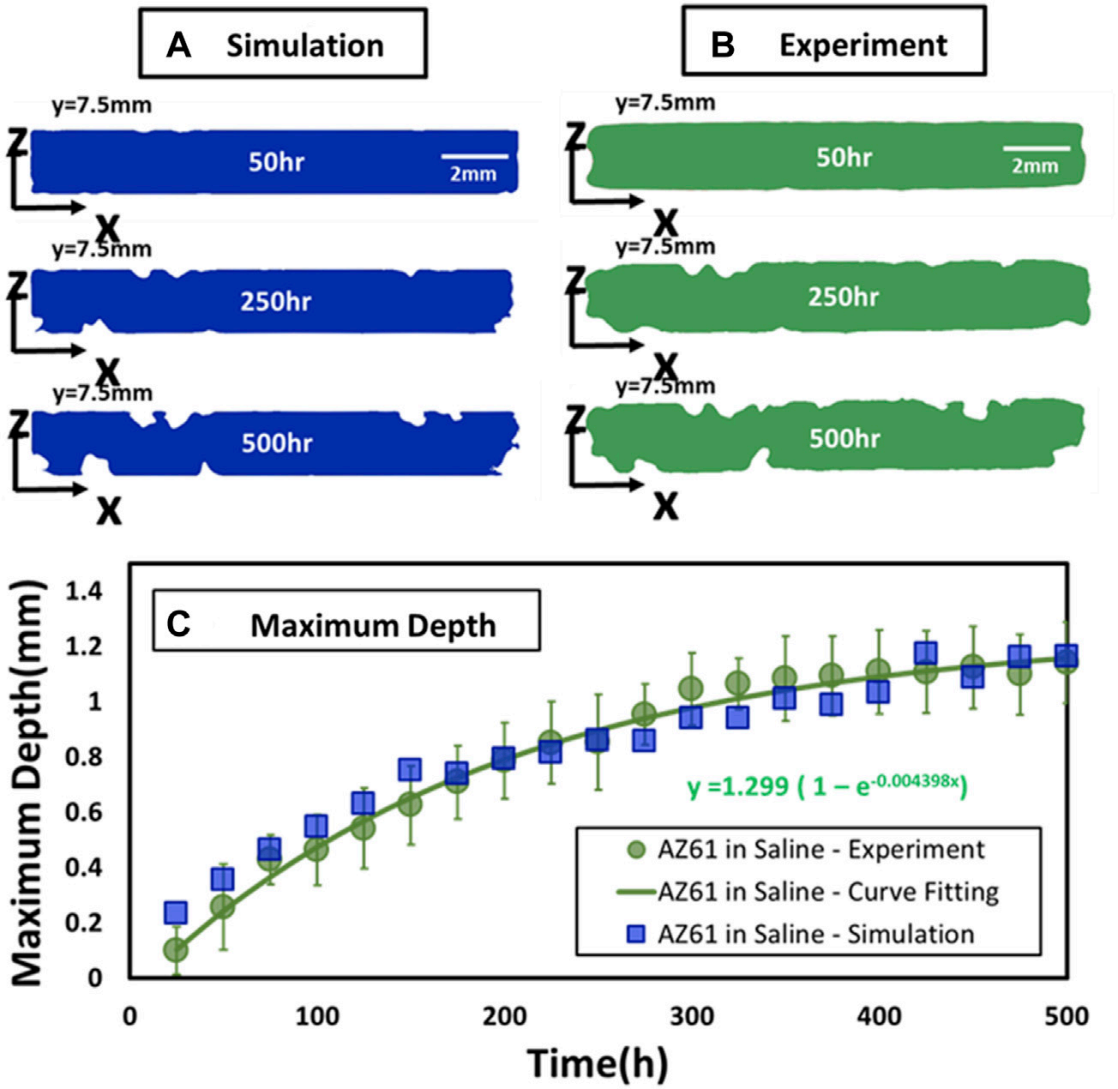
Comparison of experimental and simulated perpendicular cross sections. **(A,B)** Comparison of experimental and simulated corrosion patterns across three parallel cross sections at *y* = 7.5 mm at *T* = 50, 250, and 500 h. **(C)** Maximum depth of specimen at different corrosion times.

Figure 2 compares the corrosion simulation with experimental results of the mid-section (*y* = 7.5 mm) of a specimen for corrosion time at 50, 250, and 500 h. The experimental data are also assessed by time variations of the maximum corrosion depth. As shown in Figure 2C, the increase in the mean value with time (D_m_ indicated by a circular point) for 11 specimens can be fitted by an exponential function as follows:

$$D_{m}\mathrm{=1}\mathrm{.3}\left(\mathrm{1-}e^{\mathrm{0.0044}T}\right)$$ (4)

They are in general agreement with computational simulation depicted by square points in Figure 2C.

**FIGURE 3:**
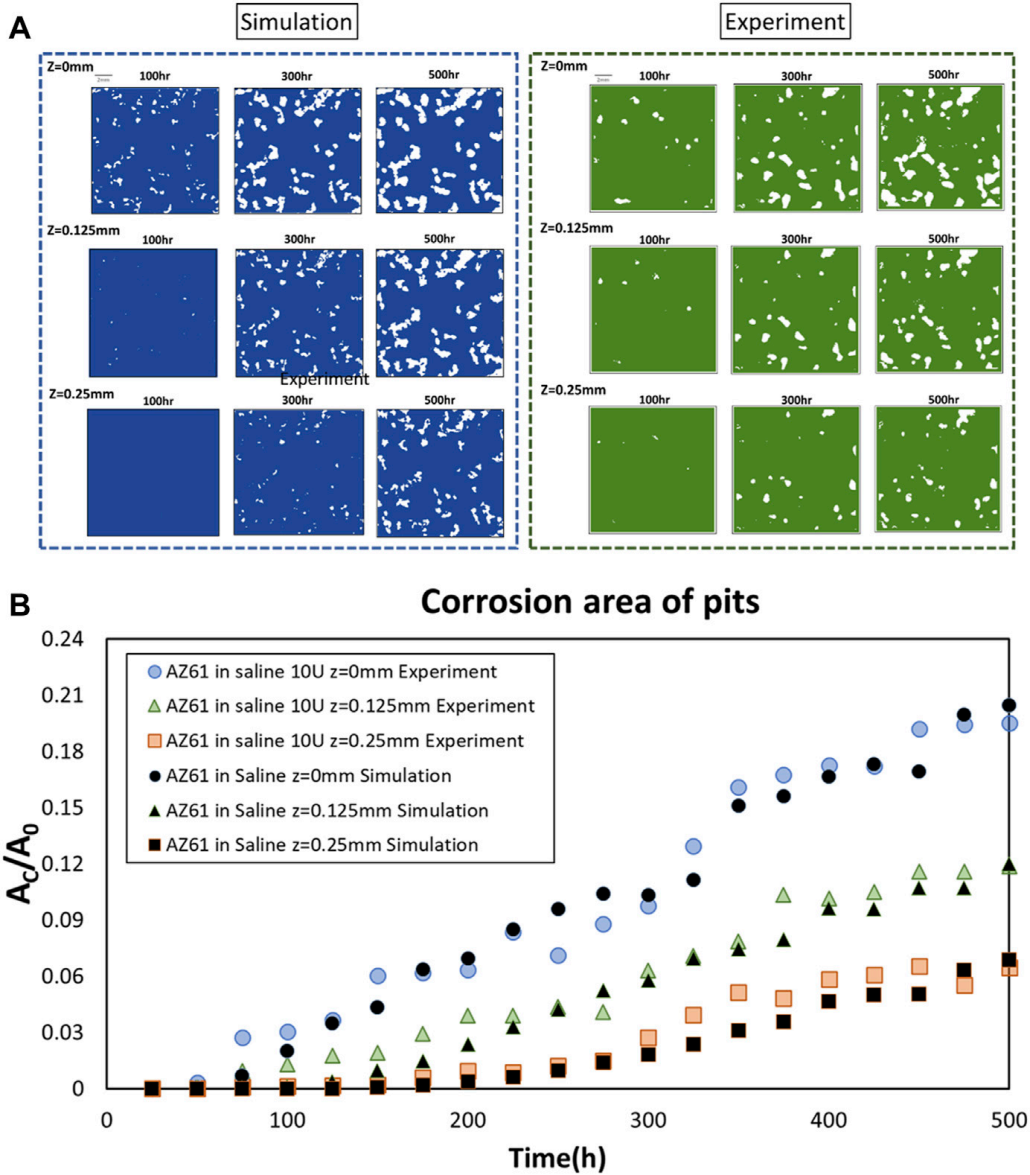
Comparison of experimental and simulated horizontal cross sections. **(A)** Comparison of experimental and simulated corrosion patterns across three parallel cross sections at *z* = 0, 0.125, and 0.25 mm at *T* = 100, 300, and 500 h, respectively. **(B)** Projected corrosion area A_C_ on three planes at *z* = 0, 0.125, and 0.25 mm.

Comparison of corrosion patterns between simulation and experimental results is presented in Figure 3A for these three planes Z = 0, 0.125, and 0.25 mm at three times *T* = 100, 300, and 500 h, respectively. The results are further analyzed by measuring the total corroded surface A_C_ on these three planes over 500 h. Figure 3B presents the mass loss per surface area (MLPA) obtained from the computational analysis and experimental measurement. Three features can be noticed in this figure:

1) The slope of A_C_/A_O_ on the surface is practically the same for experimental and computational data.
2) The slope of corrosion for the inner layer (*Z* = 0.125 mm) is much smaller than that on the surface for *T* < 250 h; they are higher than those on the mid-section (*Z* = 0.25 mm).
3) The slopes for these two inner layers continue to increase and approach those of the surface as *T* reaches 500 h.

**FIGURE 4:**
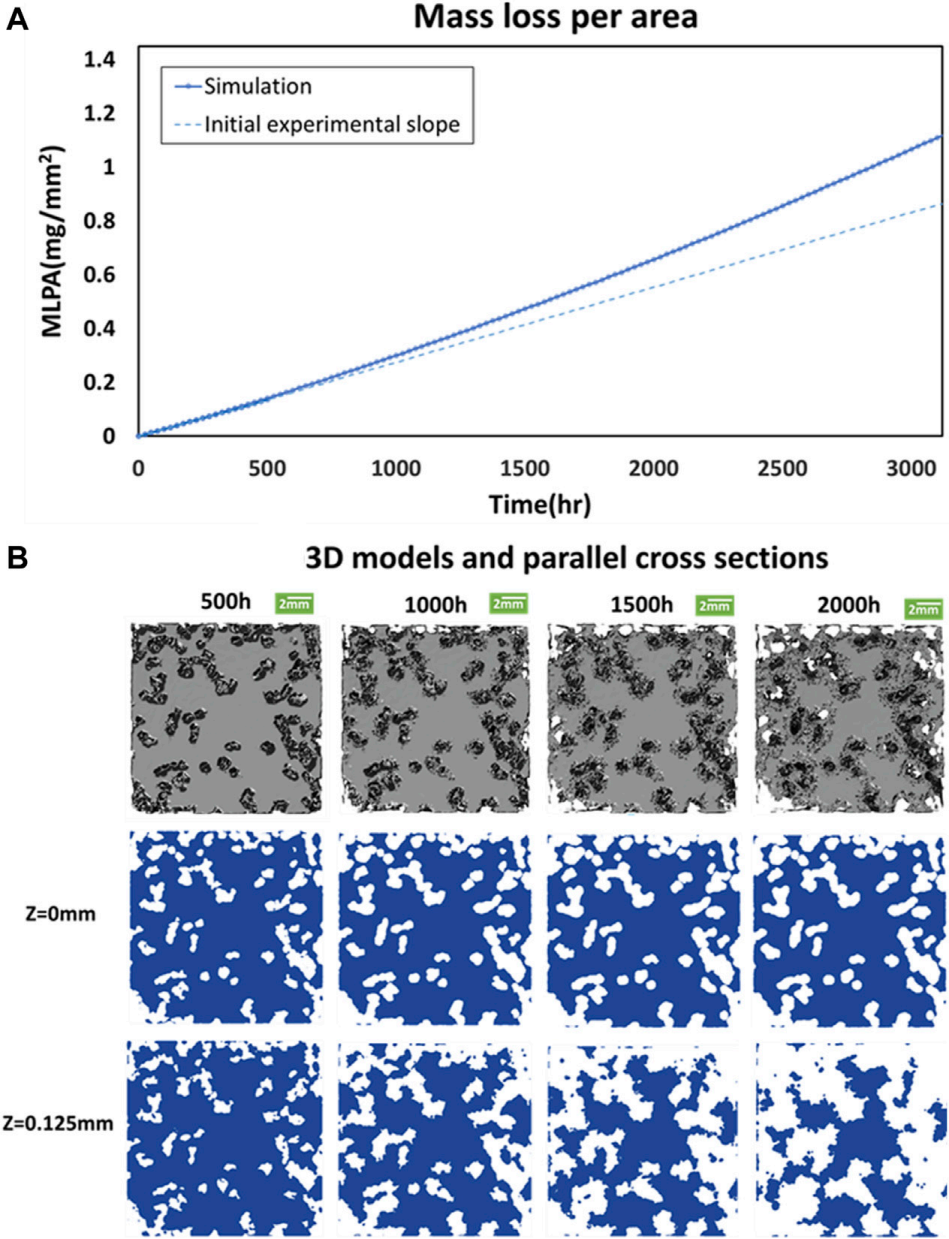
Long-term predictions. **(A)** Mass loss per area of the simulation model compares to the initial experimental slope at different corrosion times in the long-term. **(B)** 3D model and corrosion patterns across two parallel cross-sections at z = 0, and 0.125 mm at T = 500, 1,000, 1,500, and 2,000 h, respectively.

Figure 4A presents all the results of MLPA obtained by the computational model. The corrosion rate becomes higher when *T* approaches 1,000 h. The increase in mass loss exceeds the straight line, which is extended from the 500 h experimental measurement shown in Figure 1. The salient results in Figure 4B indicate that the corrosion in the inner layer (*Z* = 0.125 mm) exceeds that on the surface when T > 500 h. The phenomena appear due to the oxide surface effect on pitting corrosion. In other words, the inner layer is more prone to pitting corrosion.

## Discussion

Developing degradable implants requires controlling the corrosion rate with changes in geometry, environment, and mechanical characteristics for clinical application. The 3D corrosion simulation model appears promising to complement the experimental development of implants. It could facilitate geometric changes of the model and couple with 3D modeling of tissue growth in implant voids. The altered geometry is needed for stress and strain analysis. All the biomechanical parameters could affect corrosion pattern**s**. Also, electrochemical factors might interact with corrosion in remodeling processes. Modeling an implant weakness can be made by simulation for improvements before or during the experimental study. The effect on the oxide layer can be changed by coatings. The challenge is to determine if an implant can maintain its functional capability in the human body.

Although the non-autonomous models (mostly based on FEM analysis) are in a good framework and commercialized, they still have difficulties in boundary tracking and domain updating (Jafarzadeh et al., 2019), and several autonomous models still have many limitations for developing implants. In our semi-autonomous computational model, the effect of oxide layers on corrosion is considered and modeled by the oxide attribute (*OAi*). The computational results are in agreement with the micro-CT measurement for 20 time steps in 500 h. The computation appears effective to pair with experimental measurements, especially to project long-term corrosion patterns. Also, the 3D asymmetrical corroded morphology is practical in comparison with 2D models (Sharland, 1987; Pérez-Brokate et al., 2016).

In the overall mass loss evaluation as shown in Figure 1A, the data at 200–300 h have a slight tendency to reduce the degradation rate as the past report shows that corrosion products of magnesium decreased the corrosion rate (Lindström et al., 2002; Liu et al., 2019). However, due to the measurement and recombination errors of micro-CT, there are some fluctuations in the experimental data. In the study, we employed simple linear fitting for small data, and it seems to have a minor effect on the comparison of simulation results with experiments in the different indexes in our study. The increase in the corrosion rate in inner layers shown in Figure 3A indicates the surface effect.

Evaluation of corrosion pits is good for assessing function in medical applications (Apostolopoulos et al., 2013). In this study, the positions of the porous spots for simulation are based on experimental results after the specimen has been submerged in saline for 200 h. The fragile and loose places on the oxide layer could be identified by a more advanced method. A case-specific simulation can be accomplished when more accurate porous spots are prescribed as the initial oxide surface for the first 200 h simulation to specify *OA*_*i*_ values.

The area and the type of corrosion pit patterns are important indicators in the evaluation of medical supplies. In terms of the consistency between the simulation and experiment, the geometric features of the corrosion could have been highly related to the oxide layer. The property and structure of the oxide layer may even be the dominant factor in corrosion patterns.

Exploring the function of a medical material not only involves assessing the overall amount of corrosion but also includes a discussion of changes in geometric, local conditions and mechanical properties over time. The advantage of this simulation method is that it established a corrosion simulation for the 3D model with pit features that have the potential to be used in combination with the finite element analysis (FEA) with remodeling processes. The FE method can be used to obtain the local stress or other characteristics of the material. These stress values can be applied to the corrosion model to determine the probability of corrosion. The geometric changes due to corrosion are useful for re-evaluating the stress distribution by the FE method. FEA software is good for analyzing the mechanical (Bian et al., 2021) and electrochemical factors. These data can be implemented in a corrosion model with tissue in the remodeling process. The weaknesses of medical materials and implants such as bone implants, dental implants, and stents can be studied for optimization and improvement. Also, the oxide layer on this model can be changed to a coating layer to simulate a case of improved medical devices. This model also has the potential to simulate different composite materials such as biodegradable polymer implant made in PGA, PLA, and PLGA, and its feasibility needs further verification.

The ultimate goal is to simulate conditions that mimic the biodegradable medical material in the human body in order to better assess whether medical materials and implants in the body can still maintain their functional capability during the process of degradation to achieve the goal of developing degradable materials and implants.

## Conclusion

A simulation model for the pitting corrosion of the Mg alloy is established in this study. Pitting development is modeled by specifying the non-homogeneous oxide layer based on experimental data of the first 200 h corrosion of the specimen. Pitting corrosion is quantified by measuring the surface area developed across three sections of 11 specimens along with the maximum pitting depths. The results are well compared with those obtained by computational simulation for 500 h of the corrosion pattern. The computational mass loss is further studied from the results at 500–3,000 h. Combining a novel 3D simulation model with the experimental measurement using micro-CT for implant development is demonstrated. Proper setting of characteristics for the oxide layer is vital to computational modeling of pitting corrosion. The 3D model is useful for predicting long-term corrosion patterns and implant function. It could complement experimental efforts toward developing biomedical degradable implants.

## Data Availability

The raw data supporting the conclusion of this article will be made available by the authors, without undue reservation.

## Supplementary Material

Supplementary Figure S1 STL files of magnesium alloy specimens.

Supplementary Figure S2 Image processing procedures for quantitating the pitting characteristics.

Supplementary Figure S3 Flowchart of simulation.

Supplementary Figure S4 Experimental data of AZ61 in saline. **(A)** Number of pits. **(B)** Total projected area of pits on the surface.

Supplementary Figure S5 Comparison of results for four element sizes. **(A)** Convergence analysis by different grid sizes. **(B)** Reproducibility analysis by the same grid size.

Supplementary Figure S6 Ratio of the surface area in the experiment.
