# Supplementary figures and images for: A Computational Pitting Corrosion Model of Magnesium Alloys
Source: Front Bioeng Biotechnol. 2022 May 13;10:887444. doi: 10.3389/fbioe.2022.887444 (PMC9136027; doi:10.3389/fbioe.2022.887444)

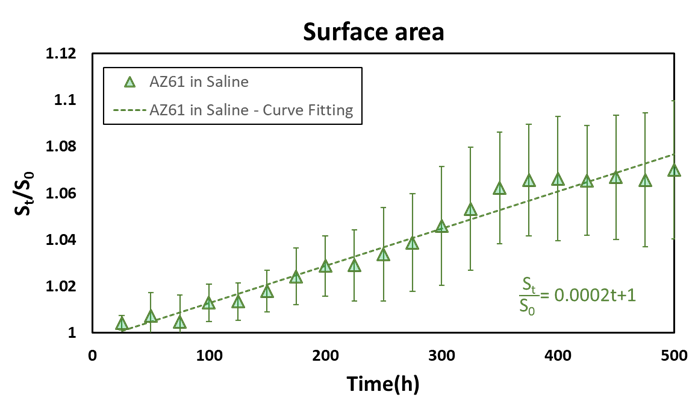

Supplement: Supplementary file 1 [file Image6.TIF]

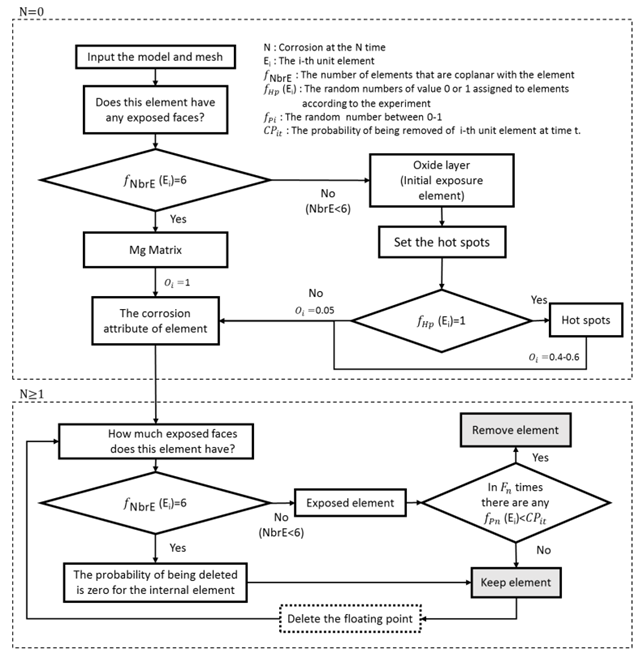

Supplement: Supplementary file 2 [file Image3.TIF]

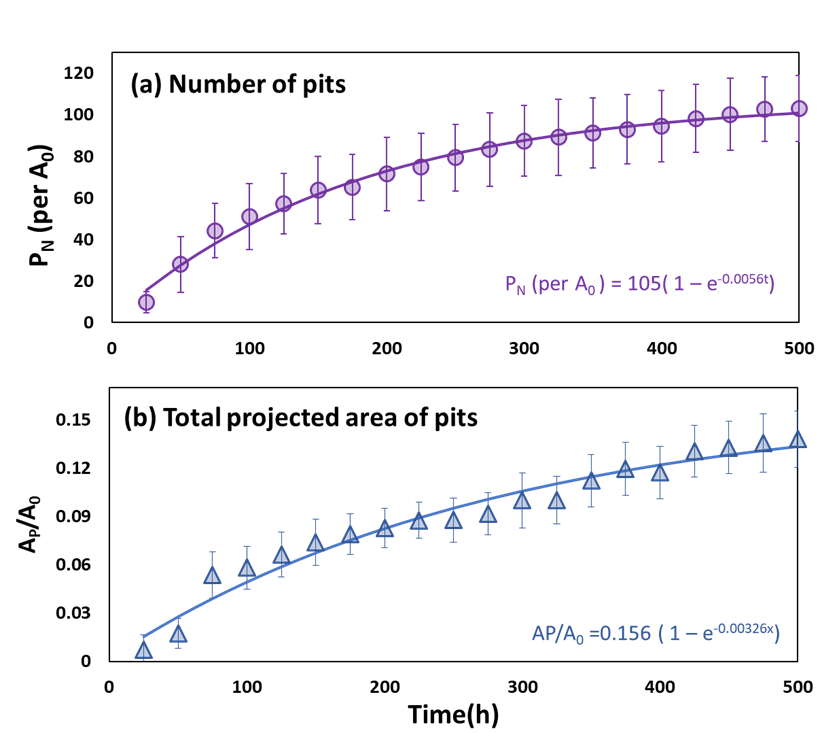

Supplement: Supplementary file 3 [file Image4.TIF]

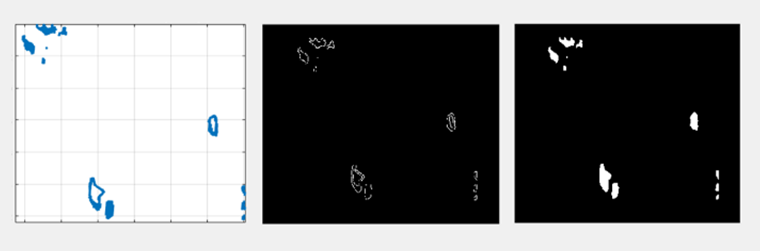

Supplement: Supplementary file 4 [file Image2.TIF]

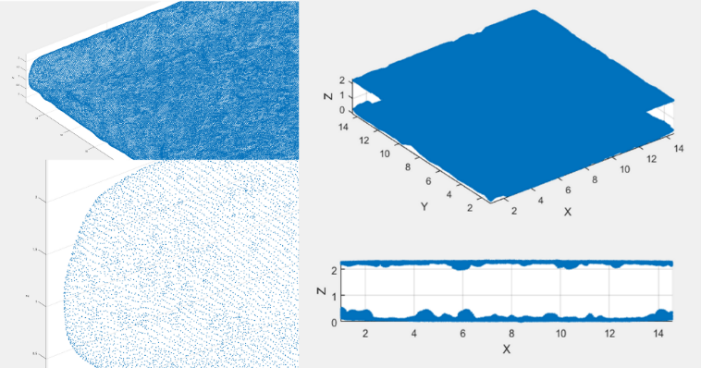

Supplement: Supplementary file 5 [file Image1.TIF]

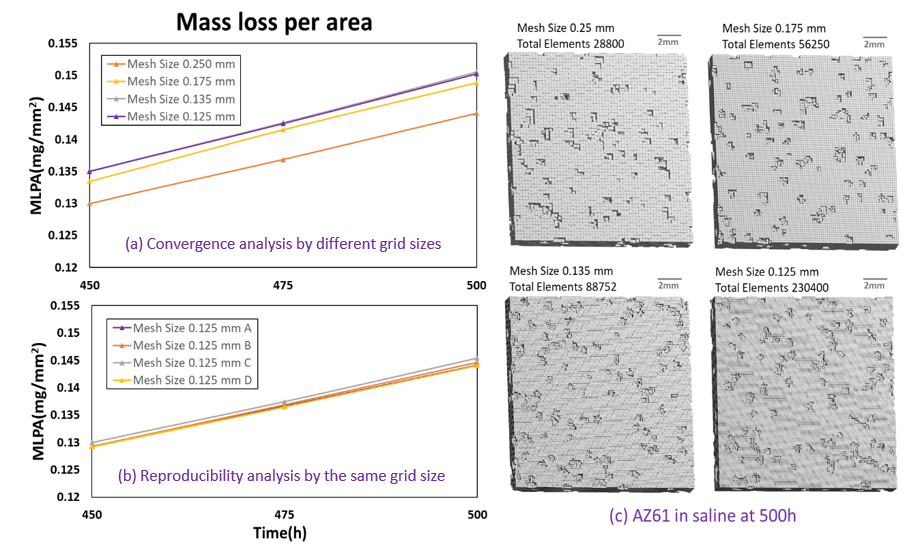

Supplement: Supplementary file 6 [file Image5.TIF]
